# Occurrence of fish species in the inland water of Murmansk Region (Russia): research in 1972-2021

**DOI:** 10.3897/BDJ.9.e68131

**Published:** 2021-05-28

**Authors:** Elena Zubova, Nikolay Kashulin, Petr Terentyev, Alexey Melekhin, Roman Konstantinovich Fedorov, Sergei Shalygin

**Affiliations:** 1 INEP KSC RAS, Apatity, Russia INEP KSC RAS Apatity Russia; 2 PABGI KSC RAS, Apatity, Russia PABGI KSC RAS Apatity Russia; 3 ISDCT SB RAS, Irkutsk, Russia ISDCT SB RAS Irkutsk Russia; 4 New Mexico State University, Las Cruces, United States of America New Mexico State University Las Cruces United States of America

**Keywords:** database, Murmansk Region, inland water, fishes

## Abstract

**Background:**

Knowledge about the distribution of organisms on Earth is important backbone of biological sciences and especially for deeper understanding of biogeography. However, much of the existing distributional data are scattered throughout a multitude of sources (including in different languages), such as taxonomic publications, checklists and natural history collections and often, bringing them together is difficult. Development of the digital storage facilities may prevent loss of important data (Ruchin et al. 2020). Project GBIF is a good example of a successful data storage facility, which allows investigators to publish biodiversity data in one safe place in one uniform format. Our dataset describes the degree of the investigation of the fish fauna of the inland water of the Murmansk Region. Murmansk Region is a Euro-Arctic Region with a heterogeneous landscape, which determines diversity of the habitats for the fish occurrence. Our dataset contains valid information about distribution of the fish species. This dataset was built upon information obtained by the members of a Laboratory of the aquatic ecosystems of the Institute of North Industrial Ecology Problems of Kola Science Center of the Russian Academy of Science (INEP KSC RAS). The dataset includes 18,509 records about 16 fish species from 14 genera (eight families) collected from 1972 to 2021. A total of 67 water bodies from 15 different basins (rivers from basins of the White and Barents Seas) was screened in order to characterise ichthyocenoses. The main purpose of publishing a database is to make our data available in the global biodiversity system to a wide range of users. The data can be used by researchers, as well as helping the authorities to manage their territory more efficiently.

**New information:**

All occurrences are published in GBIF for the first time. We would like to make this data available to everyone by adding it in the global biodiversity database (GBIF).

## Introduction

The most recent fish biodiversity study of the circumpolar Arctic has shown large knowledge gaps in species distributions and ecology (Laske et al. 2019). This fact complicates valid estimation of the tendencies of the long-term transformations of the biodiversity of the fishes in the relationships with quickly-changing environmental conditions. Extensive development of the Euro-Arctic zone of Russia, together with global environmental change, leads to the dramatic shift in structural-functional order of ecosystems. Many freshwater systems of the Murmansk Region are experiencing disturbance of the biomass production as a consequence of long-term extensive industrial pollution and imbalance of the climate. Shifting of the species composition, introduction of the invasive species and imbalance of the complex interspecies and symbiotic relationships of aquatic biota also occur (Moiseenko and Yakovlev 1990, Nost et al. 1991 Nost et al. 1997, Kashulin et al. 1999, Gidrometeoizdat 1961, Dauwalter 2000, Meltofte 2013, Laske et al. 2019, Zubova et al. 2020a). Murmansk Region is one of the largest developed and urbanised parts of the Northern-European Russia. Due to extensive pollution of the water bodies in the second half of the 20^th^ century, the focus of researchers has been on fishes as the bioindicators (Reshetnikov 1968, Reshetnikov 1980, Moiseenko 1983, Moiseenko 1991, Moiseenko 1996, Moiseenko 1997, Moiseenko 2000, Moiseenko 2002, Moiseenko and Yakovlev 1990, Nost et al. 1991, Nost et al. 1997, Lukin and Kashulin 1992, Lukin 1998, Moiseenko et al. 1994, Sharova and Lukin 2000, Kashulin et al. 1999, Kashulin et al. 2005, Kashulin et al. 2007, Kashulin et al. 2009, Moiseenko and Lukin 1999, Moiseenko et al. 2002, Kashulin 2004, Terentyev 2005, Lukin et al. 2006, Reshetnikov et al. 2011, Reshetnikov et al. 2020, Koroleva et al. 2012, Terentyev and Kashulin 2012, Zubova 2015, Zubova et al. 2015, Zubova et al. 2016, Zubova et al. 2018, Zubova et al. 2020a, Zubova et al. 2020b, Zubova et al. 2020c, Terentyev et al. 2017, Terentyev et al. 2019, Denisov et al. 2020). Fishes are an essential part of any aquatic ecosystem and as members of trophic nets, they also have a large economic value as a food source for humans. Fishes are long-living animals in the highest trophic levels of food webs of Arctic lakes. Due to their biological features, they may reflect direct and indirect changes of the environment. Study of ichthyocenoses may help to determine negative effects of the whole complex of the different factors, including the impact on the other components of the aquatic ecosystem (Moiseenko 1991, Kashulin et al. 1999). Investigation and the monitoring of the fishes as a part of the community of the inland waters of the Murmansk Region promotes determination of the tendencies of the long-term changes in the aquatic ecosystems of the Arctic zone. Results of this research also may play a role in the development of the management plans for the conservation of the species diversity of the fishes.

This study aims to describe a dataset of up-to-date data on the occurrence of fish species in inland waters of Murmansk Region (European Russia), Norway and Finland from NFH and INEP collections which we have been recently published in GBIF as the Darwin Core Archive (Zubova et al. 2021).

## Project description

### Study area description

Inland waters of the Murmansk Region, Russia

## Sampling methods

### Quality control

Each and every observation included information about the locality with geographical coordinates and date of sample collection. All these data wereobtained by the members of the Laboratory of the aquatic ecosystems of the INEP KSC RAS. From 1972 to 1989, the main contributors and researchers who identified the fishes were T. I. Moiseenko and A. A. Lukin, from 1989 to 2003 – A. A. Lukin, N. A. Kashulin and I. M. Koroleva, from 2003 to 2011 – N. A. Kashulin, I. M. Koroleva and P. M. Terentyev, from 2011 to 2021 – N. A. Kashulin, I. M. Koroleva, P. M. Terentyev and E. M. Zubova. The majority of the coordinates (1972 – 2019) were taken with the utilisation of the following Web services: Google Maps and Yandex Maps. Since 2020, coordinates were taken from the actual locations with GPS tools. The fish collection was performed by means of a set of gill-nets following specific protocol (Sandimirov et al. 2019). The species were determined using J.S. Nelson (Nelson 2006) and R. Fricke et al. (Fricke et al. 2021). All species definitions were carried out after euthanising the animals.

### Step description

The field names of the dataset were chosen according to Darwin Core (Wieczorek et al. 2012) and include the following: "collectionCode", "institutionCode", "datasetName", "basisOfRecord", "occurrenceID", "country", "countryCode", "stateProvince", "recordNumber", "sex", "lifeStage", "eventDate", "year", "month", "day", "locality", "habitat", "decimalLatitude", "decimalLongitude", "geodeticDatum", "coordinateUncertaintyInMetres", "scientificName", "kingdom", "phylum", "class", "order", "family", "genus", "specificEpithet".

## Geographic coverage

### Description

This dataset contains information about distribution of the fish species of 67 freshwater waterbodies (15 river basins) of the Murmansk Region (Table 1).

Note: reservoirs in the Basins of the Pasvik River and Tuloma River were considered as isolated water bodies; Okunevoye Lake and Semenovskoye Lake are isolated urban water bodies of Murmansk City, the hyphen denotes the uncertainty of belonging to any river basin.

Research was held within the territory of the Murmansk Region. It occupies the eastern part of the Baltic Shield, which consists of rocks of the magmatic foundation and loose quaternary sediments on the top. The total area of the Murmansk Region is 144,900 km^2^ and the majority of the area is within the Polar Circle. The geographic area of Murmansk Region is divided into two parts: continental (territory west from Kandalaksha – Murmansk conventional meridian line) and peninsular (territory east from Kandalaksha – Murmansk conventional meridian line) and Sredniy/Rybachiy Peninsula (north-western Barents Sea coast of the Murmansk Region) (Miloserdov 1971, Gidrometizdat 1968).

**Figure 1.**
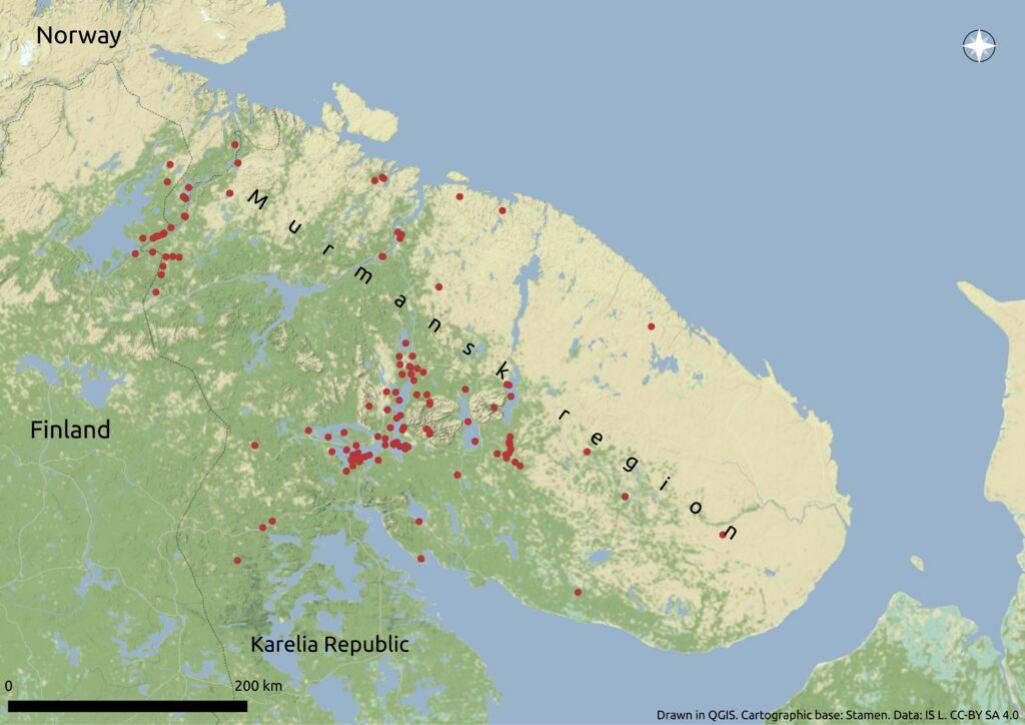
Sample sites of the fish material on the territory of the Murmansk Region.

The location of the sample sites is shown in Fig. 1. There are many large industrial manufacturers in the Murmansk Region; they are the main sources of the anthropogenic pollution of the inland water. Industrial pollution of the water bodies of the Murmansk Region is caused by operation of mining and processing of minerals, as well as transport and energy facilities.

The territory of the Murmansk Region is extremely heterogeneous. There are massive mountains (Khibiny, Lovoserskie, Chuna and Volchyi tundry) in here with the following altitude range: 900-1200 m a.s.l. (Miloserdov 1971). Terrain on the border with Finland and Norway is represented by frequent lakes within the forests, sometimes with swamps. The landscape of the region is mainly ridge-hilly (120-450 m a.s.l.). Hills and ridges are 10-60 m to 180 m a.s.l.. Specificity of the relief and climate (influence of the warm Atlantic oceanic streams, determining warm airflow from the west) determines clear vertical, meridional and latitudinal zonality, as well as mosaic distribution of the forests and tundra. Moraine, sandy and sandy loam soils with boulders and gravel (with 6 m thickness) were dominant on the investigated territory. Sand and pebble soils with boulders were distributed in the valleys of some rivers. Rubble-sandy, rocky and broken stones occurred on the slopes and tops of the mountains. Basic and ultra-basic magmatic rocks, which may be seen in cliffs of mountain slopes are usually covered by loose soils (Richter 1946, Gidrometeoizdat 1961, Miloserdov 1971, B.I. Koshechkin 1975). Magmatic rocks are here characterised by high calcium, magnesium and iron content, which give elevated buffer capacity of the water bodies (Moiseenko 1996).

### Coordinates

66.92427 and 68.850118 Latitude; 28.308064 and 39.013761 Longitude.

## Taxonomic coverage

### Description

Species cited in literary sources, but not confirmed by our catches, are not considered here. Species rainbow trout *Oncorhynchus mykiss* and carp *Cyprinus carpio* have been introduced, the other species are native. Our studies of the water bodies of Murmansk Region revealed the presence of two whitefish *Coregonus lavaretus* morphs: sparsely rakered and medium rakered. The sparsely rakered whitefish is the most common and can be found in water bodies on its own, while the medium rakered morph is less common and observed only alongside the sparsely rakered one. In general, in sparsely rakered whitefish, the number of gill rakers ranges between 15 and 31, in medium rakered whitefish between 27 and 44. Amongst whitefishes with 27 to 31 gill rakers, both sparsely rakered and medium rakered morphs were observed, distinguishable by the shape of the rakers.

### Taxa included

**Table:** 

Rank	Scientific Name	Common Name
species	*Salmo salar* Linnaeus, 1758	Salmon
species	*Salmo trutta* Linnaeus, 1758	Brown trout
species	*Salvelinus alpinus* (Linnaeus, 1758)	Char
species	*Thymallus thymallus* (Linnaeus, 1758)	Grayling
species	*Coregonus lavaretus* (Linnaeus, 1758)	Whitefish
species	*Coregonus albula* (Linnaeus, 1758)	Vendace
species	*Oncorhynchus mykiss* (Walbaum, 1792)	Rainbow trout
species	*Esox lucius* Linnaeus, 1758	Pike
species	*Osmerus eperlanus* (Linnaeus, 1758)	Smelt
species	*Cyprinus carpio* Linnaeus, 1758	Carp
species	*Phoxinus phoxinus* (Linnaeus, 1758)	Minnow
species	*Perca fluviatilis* Linnaeus, 1758	Perch
species	*Gymnocephalus cernua* (Linnaeus, 1758)	Ruff
species	*Lota lota* (Linnaeus, 1758)	Burbot
species	*Platichthys flesus* (Linnaeus, 1758)	Flounder
species	*Pungitius pungitius* (Linnaeus, 1758)	Nine-spined stickleback

## Usage licence

### Usage licence

Creative Commons Public Domain Waiver (CC-Zero)

## Data resources

### Data package title

Fishes of Murmansk Region from INEP and NFH collections

### Resource link

https://www.gbif.org/occurrence/search?dataset_key=281d50e6-3990-4a02-83b1-a6ca3330ee97&gadm_gid=RUS.45_1

### Number of data sets

1

### Data set 1.

#### Data set name

Fishes of INEP and NFH collections

#### Data format

DwC-A

#### Number of columns

29

#### Download URL

https://www.gbif.org/occurrence/download?dataset_key=281d50e6-3990-4a02-83b1-a6ca3330ee97&gadm_gid=RUS.45_1

#### 

**Data set 1.** 

Column label	Column description
id	GBIF id of record
collectionCode	Acronym for the collection, indicating the group of organisms
datasetName	Dataset name
basisOfRecord	Recommended best practice is to use the standard label of one of the Darwin Core classes
occurrenceID	An identifier for the occurrence (as opposed to a particular digital record of the occurrence)
recordNumber	Сollector number
sex	Sex
eventDate	The date-time or interval during which an event occurred. For occurrences, this is the date-time when the event was recorded. Not suitable for a time in a geological context.
year	The integer day of the month on which the event occurred
month	The ordinal month in which the Event occurred
locality	Locality (water body name etc.)
habitat	Ecology zone of water body
decimalLatitude	The geographic latitude (in decimal degrees, using the spatial reference system given in geodeticDatum) of the geographic centre of a location
decimalLongitude	The geographic longitude (in decimal degrees, using the spatial reference system given in geodeticDatum) of the geographic centre of a location.
geodeticDatum	The ellipsoid, geodetic datum or spatial reference system (SRS) upon which the geographic coordinates given in decimalLatitude and decimalLongitude are based
coordinateUncertaintyInMetres	The horizontal distance (in metres) from the given decimalLatitude and decimalLongitude describing the smallest circle containing the whole of the location
scientificName	The full scientific name, with authorship and date information
institutionCode	The name (or acronym) in use by the institution having custody of the object(s) or information referred to in the record
countryCode	The standard code for the country in which the Location occurs
stateProvince	The name of the next smaller administrative region than country in which the Location occurs
lifeStage	The age class or life stage of the biological individual(s) at the time the Occurrence was recorded
day	The integer day of the month on which the Event occurred
kingdom	The full scientific name of the kingdom in which the taxon is classified
phylum	The full scientific name of the phylum or division in which the taxon is classified
class	The full scientific name of the class in which the taxon is classified
order	The full scientific name of the order in which the taxon is classified
family	The full scientific name of the family in which the taxon is classified
genus	The full scientific name of the genus in which the taxon is classified
specificEpithet	The name of the first or species epithet of the scientificName

## Additional information

More detailed information about the biological characteristics of the fishes of the inland water bodies of Murmansk Region may be found in the online data base «L.» with the following Web address:  Biology of fish species in the inland water of Murmansk region (Zubova et al. 2021b).

## Figures and Tables

**Table 1.** List of the freshwater waterbodies of the Murmansk Region and their basins with investigated fish fauna, 1972-2021.

Name of the river’s basin	Name of the waterbody
Pasvik River basin	Ala-Nautsiyarvi Lake, Virtuovoshyaur Lake, Ilya-nautsiyarvi Lake, Kuetsyarvi Lake, Riuttikyavr Lake, Toartesyaur Lake, Shuoniyarvi Lake, Kaytakoski Reservoir, Rayakoski Reservoir, Khevoskoski Reservoir, Yaniskoski Reservoir
Lotta River Basin	Kocheyaur Lake
Tuloma River Basin	Nizhnetulomskoye Reservoir, Tuloma River
Kola River Basin	Kakhozero Lake, Kolozero Lake
Niva River Basin	Bolshoy Vudyavr Lake, Verkhnyaya Pirenga Lake, Vuekyaur Lake, Zayachye Lake, Imandra Lake, Kovdor Lake, Krugloye Lake, Kumuzhye Lake, Malyy Vudyavr Lake, Nizhnyaya Pirenga Lake, Nizhneye Chalmozero Lake, Nyudyavr Lake, Paykunyavr (Goltsovoye) Lake, Permus Lake, Pechozero Lake, Staroye Lake, Shchuchye Lake, Kuna River, Kurenga River, Pirenga River
Umba River Basin	Nizhneye Kapustnoye Lake, Umbozero Lake
Voronya River Basin	Verkhniy Tsagayavr Lake, Nizhniy Tsagayavr Lake, Lastyavr Lake, Lovozero Lake, Seydozero Lake, Sharyavr Lake, № 190 Lake, 1; № 190 Lake, 4; № 194, 3 Lake; Tsaga River
Varzuga River Basin	Verkhneye Panskoye Lake, Varzuga River, Kitsa River, Pana River
Ponoi River Basin	Makarovskoye Lake, Pesochnoye Lake, Travyanoye Lake, Sakharnaya River
Kovda River Basin	Kutsayoki River, Verman River, Voyta River
Ura River Basin	Bolshoye Uragubskoye Lake
Rosta River Basin	Bolshoye Lake
Teriberka River Basin	Dolgoye Lake, Dolgoe Lake 2 (Teriberka)
Ilyinka River Basin	Goluboe Lake
Bolshaya River Basin	Uzkoe Lake
-	Okunevoye Lake
-	Semenovskoye Lake
